# Clinical Insights Into Eating-Induced Reflex Epilepsy: A Case Report of an Eight-Year-Old Girl

**DOI:** 10.7759/cureus.67862

**Published:** 2024-08-26

**Authors:** Varshini Chandrasekhar, Rangesh Kumar Balakrishnan, Vidhyasagar K, Shreenivas Rachakonda

**Affiliations:** 1 Pediatrics, Saveetha Medical College and Hospital, Saveetha Institute of Medical and Technical Sciences, Saveetha University, Chennai, IND

**Keywords:** gastroesophageal reflux disease, focal seizures, genetic testing, anti-epileptic drugs, eating epilepsy

## Abstract

Eating epilepsy is a rare condition in children where seizures are triggered by the act of eating. An eight-year-old girl presented with seizures occurring primarily during mealtimes, characterized by a fixed gaze, jaw hypotonia, and impaired awareness. These seizures began at age seven, were initially uninvestigated, and progressively worsened over the year, reaching up to 20-30 episodes per meal. Diagnostic tests, including blood work, upper gastrointestinal endoscopy, psychiatric evaluation, and magnetic resonance imaging (MRI), were normal. The EEG showed generalized epileptiform activity, suggesting a seizure disorder, but the exact cause was unclear. After ruling out more common conditions with similar symptoms, such as gastroesophageal reflux disease, Sandifer syndrome, and psychogenic non-epileptic seizures, the diagnosis of reflex eating epilepsy was made in the end through a process of elimination, combining clinical features with EEG findings and through reviewing the literature. Treatment with oral sodium valproate monotherapy led to significant symptomatic improvement, reducing the frequency of seizures during meals.

## Introduction

Epilepsy, a condition marked by recurrent unprovoked seizures, is a complex neurological disorder that affects individuals of all ages [1]. Reflex epilepsy, a subtype of this condition, is characterized by seizures that are triggered by specific stimuli or actions [2]. Unlike unprovoked seizures, reflex seizures are often, though not exclusively, provoked by various stimuli such as somatosensory, visceral, visual, auditory, gustatory, olfactory, or cognitive factors. In reflex epilepsy, these seizures can also be triggered by complex activities such as reading, talking, or listening to music. It is also common for reflex seizures to occur alongside spontaneous seizures of similar or different types [3,4].

The term "reflex epilepsy" specifically refers to conditions where almost all seizures are consistently provoked by one particular stimulus or a defined group of stimuli. Reflex eating epilepsy, a rare form of reflex epilepsy, is triggered by the act of eating. In this condition, seizures typically occur either during or shortly after meals [5]. The process of eating involves multiple stages, from the initial thought of food to the final feeling of satiety, with seizures potentially occurring at various stages. These seizures may present as focal, either with preserved awareness or impaired awareness, though generalized-onset seizures are less common. Globally, the prevalence of eating epilepsy is estimated to be between 0.05% and 0.1% of all cases of epilepsy with a male predominance [6,7]. A notable number of these cases have been reported in South Asia, which suggests a possible genetic or ethnic predisposition, possibly influenced by specific dietary practices or eating habits prevalent in the region [7]. Management of this condition often requires a combination of anti-seizure medications, and breakthrough seizures remain common, leading to significant psychosocial distress for affected individuals and their families. This case report details the challenging diagnostic journey of an eight-year-old girl from South India, who, after presenting with sudden-onset episodic seizures while chewing and swallowing her food, was ultimately diagnosed with reflex eating epilepsy, highlighting the complexity and rarity of this condition.

## Case presentation

An eight-year-old female child, the firstborn of a non-consanguineous marriage, presented to the outpatient clinic with a history of sudden-onset episodic unawareness while eating only. She was delivered at term after an uncomplicated pregnancy and had no significant post-natal complications. Developmental milestones were found to be appropriate for age and good scholastic performance. She was immunized as per the national immunization schedule up to age. There was no family history of seizures. Upon examination, anthropometry revealed a weight of 24 kg and height of 123 cm, both falling 0 to -1 Z score as per WHO growth charts. In the general examination, there was no facial dysmorphism or neurocutaneous markers that were noted. Vitals and capillary refill perfusion were also within normal limits, and neurological examination revealed no abnormalities with normal findings. The child was found to have these episodes that were sudden in onset for the past year since the age of seven for which no prior workup was done. Her parents identified these episodes to occur during mealtime. Each seizure lasted only a few seconds and occurred shortly after starting a meal. It was marked by a fixed gaze, jaw hypotonia, and impaired awareness for about five seconds, resolving spontaneously within a few moments. As she continued chewing or swallowing her food, these seizures would resume. It can be seen in Video 1. During these episodes, she would bend forward and have a vacant stare for a few seconds before resuming her eating indicative of the seizure episode. 

**Video 1:** Seizure episode following ingestion of food

Initially, the episodes were not investigated by the parents. Over the past year, the frequency, duration, and severity of the seizures have progressively increased, from occurring once or twice a day during meals to as many as 20-30 times within a single meal. These seizures have consistently occurred only during mealtimes and not at other times of the day. A comprehensive diagnostic evaluation was conducted to explore various differential diagnoses, including gastroesophageal reflux disease, Sandifer syndrome (a condition involving gastroesophageal reflux and abnormal body posturing), torticollis, and frontal lobe lesions. Blood workup done, including complete blood count, renal function test, electrolytes, liver function test, blood sugar, and blood gas analysis, were all found to be normal. This helped rule out metabolic abnormalities contributing to the seizure episodes.

**Figure 1:**
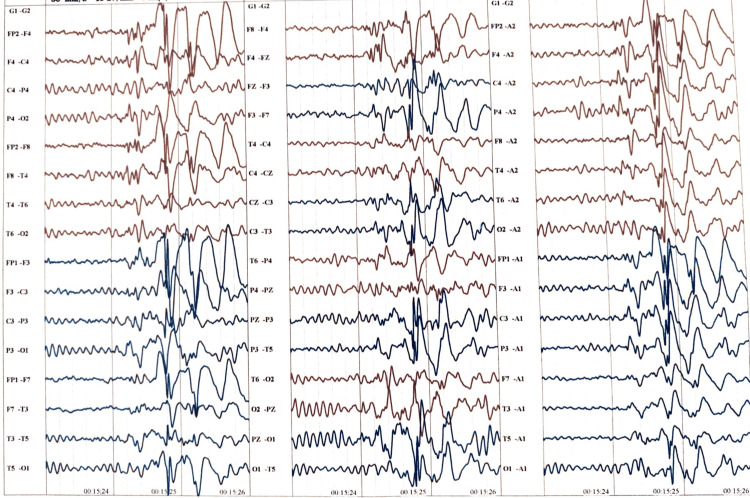
Epileptiform activity noted in EEG

Upper gastrointestinal endoscopy revealed no abnormalities related to gastroesophageal disease. Additional differential diagnoses considered included behavioral disorders, malingering, and psychogenic non-epileptic seizures. To rule out functional seizures, a detailed psychiatric evaluation was performed, including the Rorschach inkblot test. Furthermore, tests such as the Wisconsin Card Sorting Test and Wechsler Memory Scale subtests were conducted to exclude frontal lobe pathology and malingering. Conventional EEG with a 10-20 system of leads placement was done. The EEG seen in Figure 1 shows paroxysmal bilaterally synchronous spike discharges. There were no three cycles per second bisynchronous spike and wave discharges, which helped rule out absence seizures.

In addition to the psychiatric evaluation, an abnormality in the EEG aided in ruling out psychogenic non-epileptic seizures. Brain MRI, conducted to detect any cerebral malformations, was normal. Once all the common diagnoses were ruled out, we reviewed the literature once again to identify any other possible seizure, which would present with the above-mentioned findings. Given the clinical features observed during mealtimes and the EEG findings, a diagnosis of reflex eating epilepsy was made, supported by a literature review. The child was initiated on a treatment regimen of oral sodium valproate monotherapy, prescribed at a dosage of 20 mg/kg/day, divided into multiple doses throughout the day. This approach aimed to provide stable and consistent therapeutic levels of the medication. Following the start of this medication, there was a noticeable improvement in the child’s condition. The frequency of seizures decreased significantly from occurring 10-20 times per meal to approximately seven to nine times per meal. This reduction indicates that the medication effectively managed the seizures, significantly improving the child’s quality of life during mealtimes.

To ensure the continued effectiveness of the treatment and to monitor for any potential side effects, the parents were advised to schedule follow-up appointments every three months. These regular visits were essential for adjusting the medication dosage if necessary, monitoring the child’s response to treatment, and assessing any adverse effects that could arise from the medication. Adhering strictly to the prescribed medication regimen was crucial for maintaining progress and achieving optimal seizure control. Since the diagnosis and the initiation of treatment, the child has consistently attended these follow-up appointments every three months. During these visits, significant improvements in seizure control and overall symptom management have been observed. The treatment has been effective in reducing the frequency and severity of the seizure episodes. In addition to monitoring the effectiveness of the treatment, genetic testing was recommended to explore any potential underlying genetic factors that might contribute to the child’s condition. However, due to financial constraints, the parents decided not to pursue this testing. Despite this, the ongoing follow-up care has focused on optimizing the child's seizure management and ensuring continued progress with the current treatment plan.

## Discussion

Eating epilepsy, a rare form of reflex epilepsy predominantly observed in the Indian subcontinent and infrequently reported outside this region, is mainly documented in small series or isolated reports [3]. Sri Lanka reports the highest prevalence at 5.6% of all epilepsy cases, contrasting with our findings in North India where it constitutes less than one percent (0.78%, 12 out of 1,532 cases). Globally, eating epilepsy affects an estimated 0.05% to 0.1% of all epilepsy cases. There is a notably higher incidence in South Asia compared to other regions, suggesting potential genetic or dietary influences, possibly linked to rice-based diets and large meals that could trigger seizures through mechanisms such as vagal reflex due to gastric distention. Eating epilepsy typically shows a male predominance and often begins in the second decade of life [5,6]. Our study identified chewing and swallowing food as triggers for the seizures with which the child presented. Temporolimbic seizures triggered by eating suggest that complex sensory and cognitive stimuli involved in the act of eating may activate specific neural circuits in susceptible individuals, leading to seizure activity [7,8]. In previously done studies, many central nervous system malformations such as bilateral perisylvian polymicrogyria, reduced periventricular white matter bulk, mild lateral ventricle enlargement, increased subarachnoid spaces, anterior pituitary hypoplasia, dysmorphic brainstem, and midbrain hypoplasia can be seen in individuals affected with eating epilepsy. In one of the reported case studies, genetic testing revealed a heterozygous 4.8 Mb deletion spanning 15q25.3 to 15q26.1 [9]. In our case, the child had no brain malformations that were noted on MRI of the brain.

Diagnosis typically involves a thorough clinical history, EEG abnormalities, MRI, and genetic evaluation, while other conditions like gastroesophageal reflux disease and Sandifer syndrome must be ruled out [10]. Treatment often necessitates multiple anti-seizure medications, with breakthrough seizures common and contributing to significant psychosocial burden. Recognizing triggers such as chewing, swallowing, and food-related sensory cues is crucial for accurate diagnosis and effective management [11]. Approximately 30% of cases with imaging data show cortical malformations or lesions as the primary cause, indicating the origin of seizures [12,13]. The amygdala's role is pivotal in eating epilepsy due to its lower threshold for seizure activity [14]. Early identification and appropriate treatment can improve patient outcomes and mitigate the psychosocial impact of this rare epilepsy syndrome [15]. Management strategies may include dietary adjustments, tailored anti-epileptic medications, and behavioral interventions to minimize seizure frequency [16]. While 40% of cases respond well to monotherapy, approximately 60% require polytherapy for adequate seizure control [17]. Addressing the psychological and social impacts is crucial, with support from healthcare providers, families, and support groups being beneficial. Further research is needed to deepen understanding of underlying mechanisms, enhance diagnostic techniques, and develop more effective treatments [18].

## Conclusions

In conclusion, this case highlights the rare but significant occurrence of eating epilepsy in pediatric patients. The clinical presentation and EEG findings underscore the importance of considering epilepsy in the differential diagnosis of episodic seizures triggered by eating. Accurate diagnosis, comprehensive clinical evaluation including EEG monitoring, and individualized antiepileptic treatment are crucial for effective seizure management and improving the child's overall well-being. This case of reflex eating epilepsy in an eight-year-old child, marked by seizures exclusively during mealtimes, was confirmed through careful diagnostic workup and EEG results. Sodium valproate treatment was successful, markedly reducing seizure frequency and enhancing the child's quality of life. Ongoing follow-up has been essential for managing the condition and ensuring continued progress. Although genetic testing could have provided additional insights, the decision to forgo it due to financial limitations did not impede the effective management of the child's condition. This case emphasizes the need for continued research and increased awareness to advance understanding and treatment strategies for this unique form of epilepsy.
